# Cellular, molecular, and therapeutic characterization of pilocarpine-induced temporal lobe epilepsy

**DOI:** 10.1038/s41598-021-98534-3

**Published:** 2021-09-27

**Authors:** Nicholas D. Henkel, Marissa A. Smail, Xiaojun Wu, Heather A. Enright, Nicholas O. Fischer, Hunter M. Eby, Robert E. McCullumsmith, Rammohan Shukla

**Affiliations:** 1 Department of Neuroscience, College of Medicine and Life Sciences, University of Toledo, 3000 Arlington Avenue, Room 182, Toledo, OH 43614 USA; 2 Neuroscience Graduate Program, University of Cincinnati, Cincinnati, OH 45267 USA; 3 Bioscience and Biotechnology Division, Lawrence Livermore National Lab, Livermore, CA 94550 USA; 4 ProMedica, Neurosciences Institute, Toledo, OH 43606 USA

**Keywords:** Target identification, Data integration, Gene ontology, Genome informatics, Drug discovery, Neuroscience, Systems biology

## Abstract

Animal models have expanded our understanding of temporal lobe epilepsy (TLE). However, translating these to cell-specific druggable hypotheses is not explored. Herein, we conducted an integrative insilico-analysis of an available transcriptomics dataset obtained from animals with pilocarpine-induced-TLE. A set of 119 genes with subtle-to-moderate impact predicted most forms of epilepsy with ~ 97% accuracy and characteristically mapped to upregulated homeostatic and downregulated synaptic pathways. The deconvolution of cellular proportions revealed opposing changes in diverse cell types. The proportion of nonneuronal cells increased whereas that of interneurons, except for those expressing vasoactive intestinal peptide (Vip), decreased, and pyramidal neurons of the cornu-ammonis (CA) subfields showed the highest variation in proportion. A probabilistic Bayesian-network demonstrated an aberrant and oscillating physiological interaction between nonneuronal cells involved in the blood–brain-barrier and Vip interneurons in driving seizures, and their role was evaluated insilico using transcriptomic changes induced by valproic-acid, which showed opposing effects in the two cell-types. Additionally, we revealed novel epileptic and antiepileptic mechanisms and predicted drugs using causal inference, outperforming the present drug repurposing approaches. These well-powered findings not only expand the understanding of TLE and seizure oscillation, but also provide predictive biomarkers of epilepsy, cellular and causal micro-circuitry changes associated with it, and a drug-discovery method focusing on these events.

## Introduction

Epilepsy is one of the most prevalent neurological disorders and a common end point of several brain pathologies^1^. Diagnosed as the presence of spontaneous and recurrent seizures, epilepsy is a network-level phenomenon involving changes in neuronal activity, content, and density, leading to re-entrant activation of neurons embedded within brain circuits^2,3^. Although it is usually acquired following injury to a previously normal brain, familial forms of epilepsy are also known^4^.

Temporal lobe epilepsy (TLE) is the most common form of epilepsy, accounting for > 40% of adult cases^5,6^. Animal models that mimic TLE are presently available and, since their establishment, significant progress has been achieved in the understanding of epilepsy^7^. The knowledge gained through these models allows a better understanding the highly dynamic processes of TLE, such as hyperexcitability and pharmacoresistance^8^, mostly through electrophysiological techniques, and has helped reduce the number of seizures, although these cannot be completely prevented^9^. Molecular studies complementing the electrophysiological findings have revealed new molecular targets, identified antiepileptic drug (AED) candidates^10^, and confirmed experimental findings implicating the gamma-aminobutyric acid-(GABA)ergic system and ion channels in epilepsy^11^. However, the use of this information in clinical practice is impeded by the lack of studies tracing the cellular origin of these molecular changes and cell-specific drug effects in TLE^12^. Furthermore, existing molecular studies lack discrimination between the upstream (causal) and downstream (consequential) changes^13^ associated with epileptogenesis and information on their interactions and cellular correlates.

Here, we hypothesized that (1) an integrated analysis of transcriptomics data from bulk and single-cell RNAseq experiments will aid in tracing the cellular origins of TLE, (2) a Bayesian inference-based network biology approach applied to transcriptomics profiles will help identify distinct causal and consequential changes associated with TLE, and (3) drug-based transcriptomic signatures serve as a proxy for the lack of interventional data required to support causal inferences. We identified the key nodes involved in TLE and cellular correlates of several epilepsy-inducing and antiepileptic drugs.

## Results

### Gene expression changes in the epileptic phenotype

**Figure 1:**
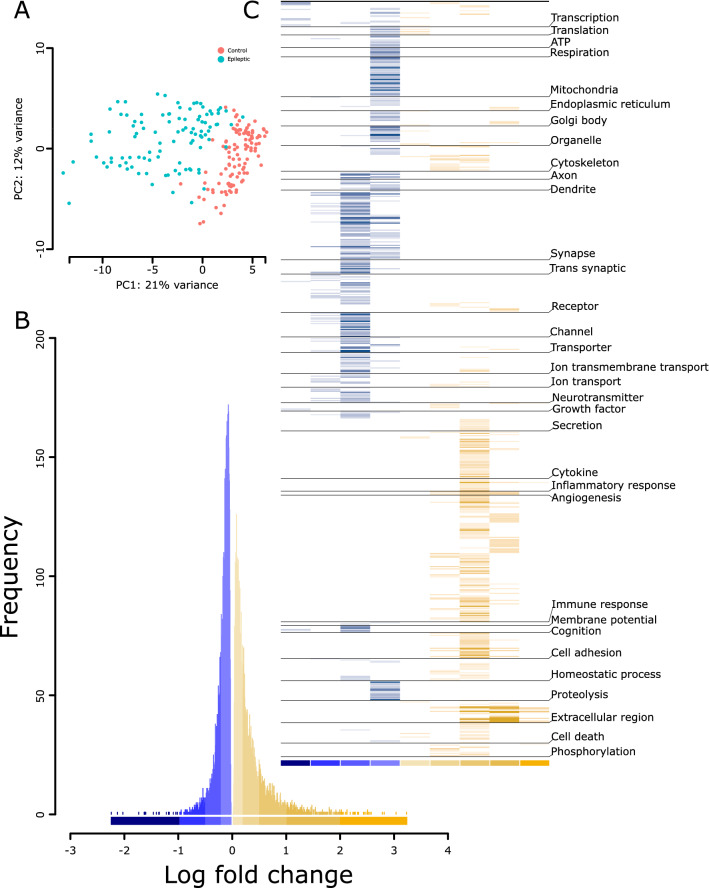
Distinct transcriptomics and pathway profiles of pilocarpine induced TLE in mice. (**A**) Principal components analysis segregating the transcriptomics profile of the control and pilocarpine induced TLE mice. (**B**) Frequency of differentially expressed genes binned based on log-fold changes across down- (blue) and up- (yellow) regulated genes. See Table S1 for genes under each bin. (**C**) Pathway profiles of up- and downregulated genes by log-fold change bin (from **B**) using Gene Ontology. The − log_10_(q value) of each pathway was used to plot the heatmap. The lighter-to-darker shades of blue and yellow indicate increasing significance of down- and upregulated pathways, respectively. See Table S2 for the pathways grouped under each theme.

To analyze changes in gene expression associated with pilocarpine-induced TLE, we used RNAseq data generated from the hippocampus of 108 pilocarpine-induced epileptic and 103 control littermates adult male Crl:NMRI (Han) FR mice (> 12 weeks)^14^. Epileptic mice displayed status epilepticus and showed spontaneous recurrent seizure. Principal component analysis clearly segregated the control and epileptic phenotypes (Fig. 1A). Indicative of the penetrant nature of the epileptic phenotype, ~ 75% (11,580/15,300) of the genes in our analysis were differentially expressed in the epileptic vs. control condition (up: 5722 genes; Down: 5858 genes; FDR < 0.05; Table S1). Biologically significant changes can occur at any level of fold change^15^, and the number of samples in the present dataset provided sufficient power to detect subtle yet significant (q < 0.05) changes. Thus, to determine the abundance of genes at different fold changes, we binned and plotted the differentially expressed genes (DEGs) at different thresholds of log-fold change (lfc) (Fig. 1B). The histogram suggests that subtle changes with an absolute lfc of < 0.2 were most abundant for both up- and downregulated changes; however, they were more prominent in the downregulated direction (down: 4156; up: 2830). Conversely, distinct changes with an absolute lfc of > 1 were few among up- and downregulated changes but were more prominent in the upregulated direction (up: 269; down: 20).

### Down- and upregulated changes in the epileptic phenotype are biased toward specific biological effects

We explored the biological pathways associated with different bins of DEGs. All identified pathways (q < 0.05) were clustered based on functional themes (Fig. 1C; Table S2).

#### Downregulated pathways

Highly downregulated genes (lfc <  − 1) corresponded to a few pathways and were associated with themes related to transcription, membrane potential, receptors, and growth factors. Moreover, 8/13 pathways in this category predominantly featured transcription factor binding, suggesting an overall decrease in regulatory events. Intermediately downregulated genes (lfc, − 1 to − 0.5) were largely related to neuronal signaling. Furthermore, 45 pathways in this bin were associated with synapse, receptors, channels, ion transport, neurotransmitter, and G-protein-coupled receptors. Subtly downregulated changes (lfc, − 0.5 to − 0.2) had 180 pathways and, like the prior bin, were predominantly associated with synapse- and signaling-related themes. The most subtle changes (lfc, 0 to − 0.2) had 174 pathways associated with ATP production, mitochondria, proteolysis, transcription, and translation, indicating impairment in energy balance and basic cell functionality in TLE. Interestingly, signaling-associated themes (axon, dendrite, synapse, receptors, ion channels, ion transport, neurotransmitter, and membrane potential) were dominant in all the downregulated pathway bins, suggesting the altered and widespread impact of neuronal activity in TLE.

#### Upregulated pathways

Highly upregulated genes (lfc > 2) corresponding to ten pathways were almost exclusively related to extracellular matrix. Other pathways in this bin were associated with phosphorylation, growth factors, and inflammation. Genes with a lfc of 1–2 corresponded to 68 pathways largely related to immune system, while also featuring extracellular matrix, angiogenesis, and organelle activity (including that of Golgi bodies and endoplasmic reticulum). Intermediately upregulated genes (lfc, 0.5–1) corresponding to 232 pathways were largely enriched in immune system processes and extracellular matrix. Further, we identified apoptosis-, homeostasis-, cell adhesion-, and cytoskeleton-associated pathways. Subtly upregulated changes (lfc, 0.2–0.5) shared most themes with the prior bin (0.5–1). There were very few most subtly upregulated pathways (lfc, 0–0.2; n = 17), which were related to transcription, translation, organelles, cytokine production, and apoptosis.

Overall, the down- and upregulated changes in the epileptic phenotype were biased toward specific biological effects. The downregulated pathways were mostly associated with intracellular and cell-to-cell signaling, while the upregulated pathways were mostly associated with the extracellular matrix, immune response, and homeostatic changes.

### TLE is associated with subtle molecular changes

Not all DEGs contributed to or informed about the epileptic phenotype. To identify epilepsy-predicting genes, we deployed a sparse classifier that used a minimal set of variables (genes) to predict TLE. Using 119 genes (Table S3; Up: 59 and Down: 60; termed “predictive gene set” hereafter), the classifier could distinguish the withheld control and epilepsy samples with 98% accuracy. For validation, we analyzed their enrichment in gene sets associated with various disease phenotypes using the Enrichr database^16^. The strongest signal stemmed from genetic reflex epilepsy (q = 6.53 × 10^−37^), epileptic encephalopathy (q = 1.5 × 10^−35^), and familial TLE (q = 4.4 × 10^−8^).

**Figure 2:**
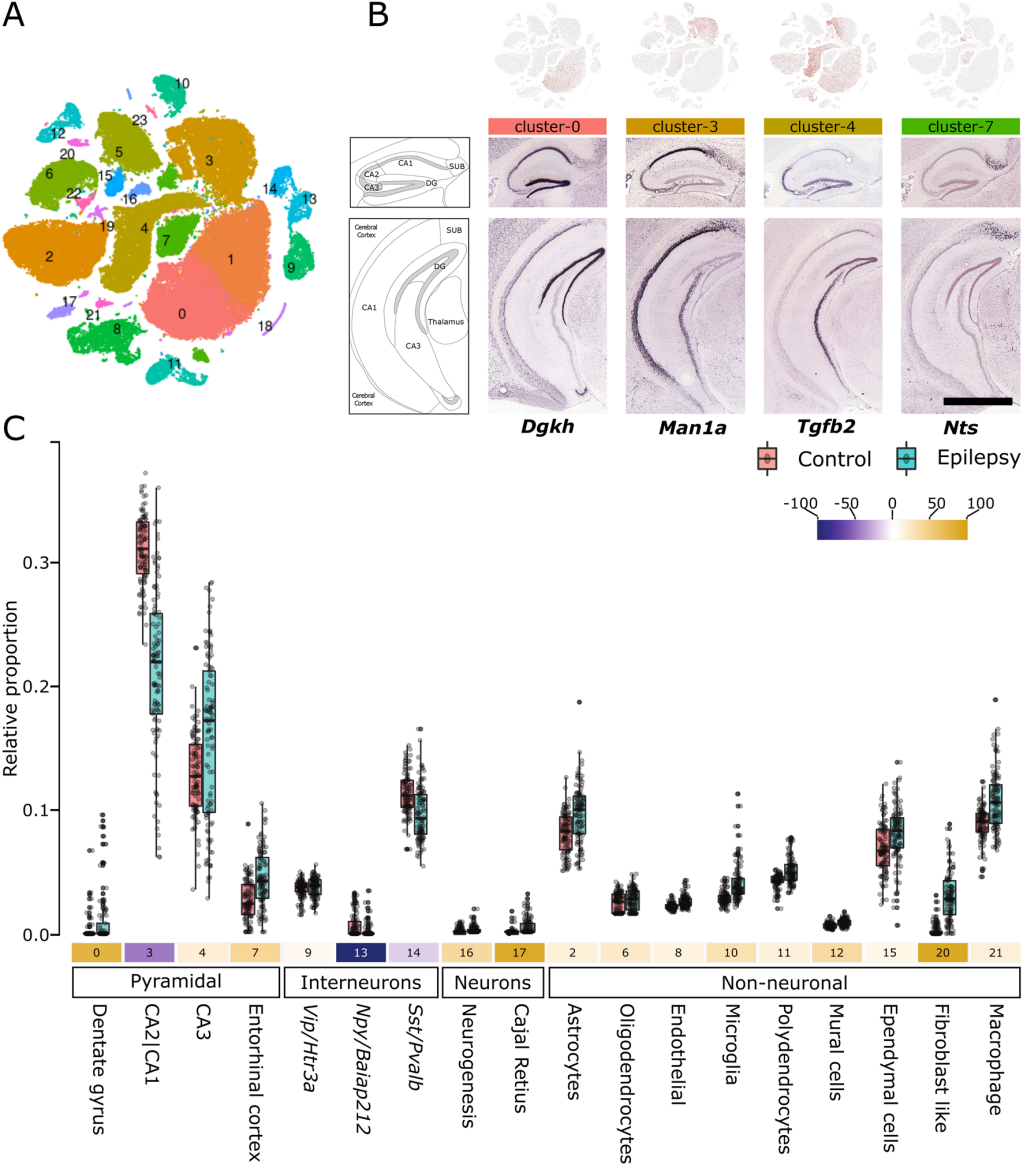
Cell-type deconvolution of TLE RNAseq data reveals altered neuronal and non-neuronal cell proportions. (**A**) Clustering of mouse hippocampus single-cell RNAseq data from Gene Expression Omnibus (GSE116470). See Figure S1 for cluster characterization and Table S4 for cluster-specific genes. (**B**) Cell-type clusters representing pyramidal neurons were enriched in different hippocampal regions and subfields. In situ hybridization data (Allen brain atlas) of genes (discrete markers, Table S4) representative of the pyramidal-neuron-specific cluster-0 (Dgkh), cluster-3 (Man1a), cluster-4 (Tgfb2), and cluster-7 (Nts) are shown. Scale bar, 1678 micron. (**C**) Proportional differences of cell-type clusters in the control and epileptic cohorts. The Y-axis shows the relative proportion of different cell-type clusters. The number below each box plot represents the cluster number from (**A**), and the colors in shades of blue and yellow indicate a decrease or increase in the percentage of relative cell proportions, respectively. All changes in proportions were significant (P < 0.001; Wilcoxon test). See Table S4for additional details.

To further characterize the predictive genes, we looked for its enrichment in (1) different DEG bins (Fig. 1B), (2) GO terms, and (3) hippocampus cell types/subfields (Fig. 2A). Within different DEG bins, the predictive genes were enriched in up- and downregulated bins of subtle (Down: lfc =  − 0.5– − 0.2, P < 0.3 × 10^−32^; and Up: lfc < 0.2, P < 0.0017; lfc = 0.2–0.5, P < 0.0013) to moderate (lfc =  − 1 to − 0.5, P < 0.90 × 10^−3^) lfcs. For GO terms (Table S3), similar to DEG bins (Fig. 1C), the downregulated predictive genes were enriched in pathways associated with cell-to-cell signaling involving both presynapse (q < 1.86 × 10^−18^) and postsynapse (q < 2.20 × 10^−21^), whereas the upregulated predictive genes were enriched in pathways involving neurogenesis (q < 3.03 × 10^−8^), regulation of gliogenesis (q < 5.23 × 10^−3^), and cellular homeostasis (q < 5.79 × 10^−4^). Finally, within the hippocampal cell types/subfields (Fig. 2, detailed in the next section), the downregulated predictive genes were enriched in cells of the dentate gyrus (cluster-0, q = 6.8 × 10^−09^) and pyramidal neurons of the cornu ammonis 1 (CA1)|CA2 (cluster-03, q = 3.60 × 10^−28^) and CA3 (cluster-04, q = 6.94 × 10^−07^) subfields, whereas upregulated predictive genes were enriched in the dentate gyrus (cluster-0, q = 7.28 × 10^−07^), astrocyte (cluster-02, q = 4.31 × 10^−26^), and pyramidal neurons of CA1|CA2 (cluster-03, q = 6.3 × 10^−3^) subfields.

Overall, narrowing down the highly informative DEGs using a sparse classifier suggested that subtle-to-moderate fold changes in the expression of a few genes enriched in the astrocytes and pyramidal cell types of the dentate gyrus and hippocampal subfields, can predict various forms of epilepsy.

### TLE is associated with altered neuronal and nonneuronal cell proportions

Although the present dataset (derived from a tissue homogenate) could assess the biological changes associated with TLE, the variability in relative cell proportions and associated biological signals was masked. Single-cell transcriptomics datasets and statistical deconvolution methods can be used to address these issues^17^. Using a single-cell transcriptomics dataset for the hippocampus^18^, we first generated 24 cell-type-specific clusters (Fig. 2A), which were characterized in two stages: (1) globally, based on the presence of known markers associated with different cell types (Figure S1), and (2) locally, based on the top upregulated DEGs associated with the respective cluster. For instance, Vip/Htr3a-, Npy/Baiap212-, and Sst/Pvalb-positive interneurons represented the top DEGs in clusters 9, 13, and 14, respectively (Figure S1). For pyramidal neurons, the clusters also corresponded to recognizable areas of the hippocampus. For instance, clusters 0, 3, 4, and 7 belonged to the dentate gyrus, CA2|CA1 subfields, CA3 subfield, and entorhinal cortex of the hippocampus, respectively (Fig. 2B). Using a support-vector-machine-(SVM)-based method^19^, highly discriminative markers for each cluster (Figure S2; Table S4) were used to deconvolve their relative proportions in the control and epileptic bulk RNAseq samples. Significant differences in proportion were detected for 17 clusters (Fig. 2C), and interesting patterns of changes were noted. First, all nonneuronal cells showed an increased relative proportion in epilepsy vs. control samples. Second, neuronal cells showed both an increase and a decrease in relative proportion. In particular, pyramidal neurons, with the exception of those belonging to the CA2|CA3 subfield, showed an increase in relative proportion, and interneurons, with the exception of Vip/Htr3a-positive ones, showed a decrease in relative proportion. Third, pyramidal neurons of the CA1|2|3 subfields showed the highest variation among all cell types.

**Figure 3:**
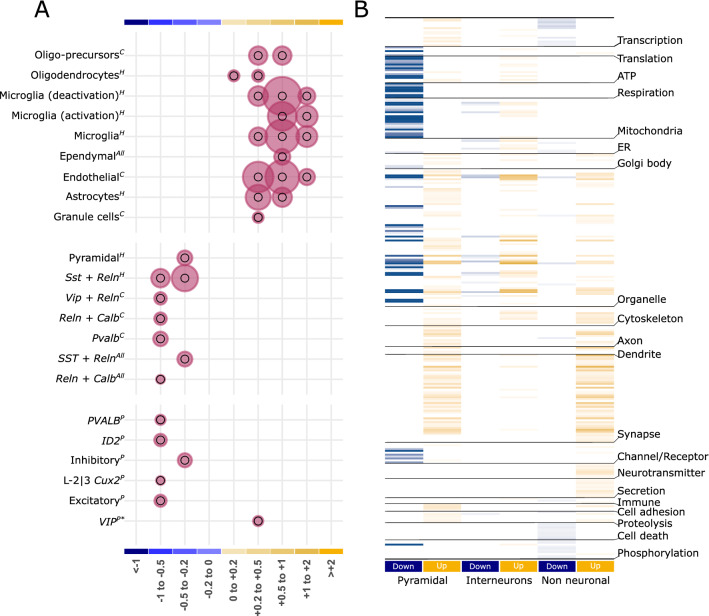
Validation of the cell-type deconvolution (**A**) and segregation of pathways affected by different cell classes (**B**). (**A**) Enrichment of cell-specific markers from an independent study within the log-fold change bins of differentially expressed genes. Note that enrichment does not provide detailed segregation of cell type, as observed in Fig. 2C. However, reflecting the increased proportion of non-neuronal cells, their enrichment is observed in the bins associated with upregulated genes. The size of the circles is proportional to the − log_10_ value of the enrichment q value. Inner black circles represent the reference representing (q value = 0.05). Significant results (q value < 0.05) are plotted exclusively. Superscripts C, H, and All represents cell specific markers from cortex, hippocampus, and all brain region obtained from Mancarci et.al.; Superscripts P represents cell-specific markers from human TLE studies obtained from Pfisterer et.al. *For Vip-interneurons, to emphasize the similarity of increased proportion, p value < 0.05 is considered, (**B**) Pathways and themes (same as Fig. 1C) affected by cell-type clusters representing pyramidal, interneuron, and non-neuronal cells. The log_10_(q value) of each pathway was used to plot the heatmap. The lighter-to-darker shades of blue and yellow indicate increasing significance of down- and upregulated pathways, respectively. See Table S5 for additional details.

We used several approaches to validate the deconvolution analysis. First, we deconvolved the data using alternative methods (Figure S3), implementing non-negative least square (NNLS)^20^, reweighted least square (RLS)^21^, and linear mixed model (LMM)^22^ based algorithms. The RLS showed the highest mean correlation of ~ 0.7 with the SVM based deconvolution performed above (Fig. 2C). Notably, the deconvolved proportions were comparable to those obtained using the SVM approach (Figure S3). Second, we performed cell-type enrichment—an approach similar to deconvolution (Fig. 2C), requiring cell-specific marker genes which can be used as a proxy to deduce cell-type proportion at lower resolutions^23^, within each DEG-bins using reported hippocampal cell-specific markers^24^ (Fig. 3A). Confirming the changed proportions, we observed the enrichment of interneurons and nonneuronal cells in down- and upregulated bins, respectively. Third, we looked at recent study using TLE single-nucleus RNAseq^11^; although the study was conducted in cortex, corroborating with our findings the directionality of changes in proportion for various neuronal subtypes was preserved. For instance, consistent with our finding (Fig. 2C), we observed an enrichment (p value < 0.03) of Vip-interneurons specific signatures from this study in the up-regulated bins (Fig. 3A). Furthermore, the enrichment of nucleus-specific signatures within each DEG bin was similar to the one observed for hippocampal cell-specific markers.

To identify genes that are affected by the altered pyramidal neurons, interneurons, and nonneuronal cells, we regressed out the variability associated with these cell proportions from the differential expression analysis. We found that 1749 (up: 709, and down: 1040), 2352 (up: 1141, and down: 1211), and 1510 (up: 761, and down: 749) genes showed a further reduced P-value after regressing the pyramidal neurons, interneurons, and nonneuronal cell proportions, respectively (Table S5). Mapping the themes observed in the prior analysis (Fig. 1C) to the pathways associated with these genes (Fig. 3B; Table S5) showed that pyramidal neurons prominently influenced the downregulation of bioenergetics (ATP, respiration, and mitochondria)-, organelle-, receptor/channel-associated pathways and consistent with the recent snRNAseq report, an upregulation of synaptic functions; interneurons affected the downregulation of organelle (mitochondria and endoplasmic reticulum)- and the upregulation of cytoskeleton-related pathways, at a low level, whereas nonneuronal cells affected the downregulation of transcription-, cell-death-, and phosphorylation-, and upregulation of cytoskeleton-, axon-, dendrite-, and neurotransmission-related pathways (Fig. 3B).

Together, deconvolving the cellular origin of gene expression consistent with the penetrant nature of TLE revealed alterations in all cell types. Opposing changes were observed in neuronal and nonneuronal cells. All nonneuronal cells exhibited an increased proportion, interneurons (except for those expressing Vip/Htr3a) showed a decreased proportion, and pyramidal neurons (particularly in CA subfields) showed the highest variation in proportion.

### Investigating causality using a Bayesian gene network analysis

**Figure 4:**
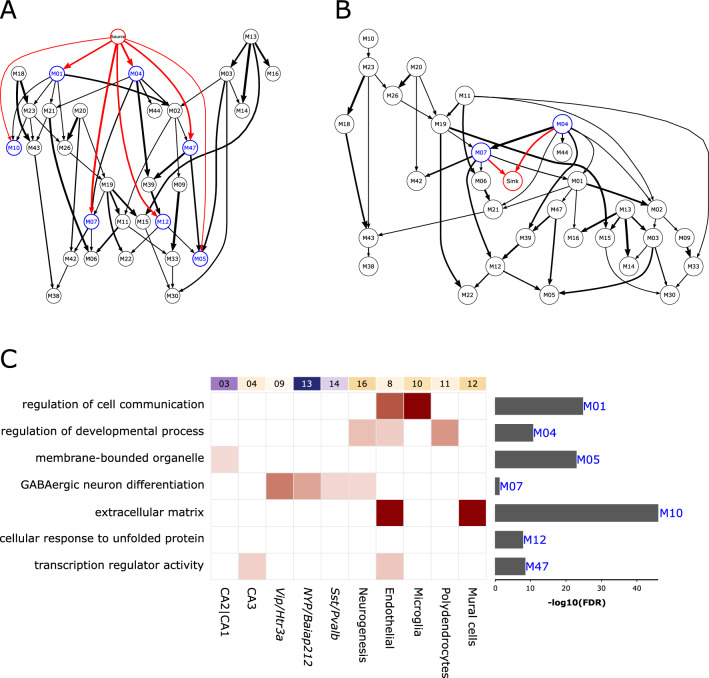
Prioritizing the TLE-associated upstream (putative causal) and downstream (putative consequential) gene modules using a Bayesian network. (**A**, **B**) Directed acyclic graph obtained after fitting the Bayesian network to the eigengenes of consensus WGCNA modules, shown as nodes. The edges represent probabilistic dependencies between the nodes from early to later (referred as parent-to-child) associations. Source and sink nodes constitute a categorical variable represented by sample labels (i.e., control and epileptic). Source node (**A**) has no parent node but has child nodes representing downstream consequential gene modules connected to it (in blue with red edges). In turn, the sink node (**B**) has no child nodes but has parent nodes representing upstream causal gene modules connected to it (in blue with red edges). Note that the M04 and M07 nodes are associated with both source and sink nodes. The thickness of the edges corresponds to the number of times (in percent) the edges were detected in the 1000 permutations used to generate the Bayesian network. (**C**) Characterization of source- and sink-associated nodes using gene ontology (GO) and cell type (from Fig. 2). Enriched GO terms are shown on the left, with significance shown as a bar graph on the right. Enrichment of cell-type clusters (top labels, same as Fig. 2C) in genes of up- and downstream nodes. The lighter-to-darker shades of pink indicate increasing significance of cell-type enrichment.

Although pathway and deconvolution analyses provided biological and cellular insights, they did not provide information about events occurring up- and downstream of the disease state. Therefore, we summarized the expression profiles of control and epileptic samples into consensus modules of co-expressed genes using consensus WGCNA^25^ and then used the module eigengene to construct a Bayesian-network modeling the probabilistic dependencies between modules as directed acyclic graphs^26^ (DAGs; Fig. 4A,B; the modules in DAG are termed “nodes” hereafter). To infer the TLE-associated up- and downstream events, we anchored a binary variable associated with sample labels (i.e., control and epileptic) to the DAG. By design, this variable served as (1) a source, with no parent node above it and the child nodes serving as downstream events, or (2) a sink, with no child nodes below it and the parent nodes serving as upstream events.

Fifty consensus modules were identified that contained 21–1575 genes. Of these, 33 were significantly enriched for at least one GO functional category (Biological-Process, Molecular-Function, or Cellular-Component) at FDR < 0.05 (Table S6 and were used to create the source and sink DAGs (Fig. 4A,B). The source node (Fig. 4A) served as the root of the source DAG and, in the order of association probability (lowest to highest), were parent to the following nodes: M05, enriched (Fig. 4C, bar graphs) with membrane-bound organelle; M10, enriched with extracellular matrix; M12, enriched with cellular response to unfolded protein; M47, enriched with transcription regulator activity; M04, enriched with regulation of developmental process; and M07, enriched with GABAergic neuron differentiation. The sink node (Fig. 4B) was connected to two parent nodes (M04 and M07), which also served as child nodes in the source DAG (Fig. 4A). In both DAGs, the M04 and M07 nodes had a parent and child relationship, respectively.

Subsequently, we looked for the enrichment of the discriminant markers of 21 cell-type clusters (Figure S2, Table S4) in the up- and downstream nodes (Fig. 4C, boxes). M04 and M07 nodes were found in both the source and sink DAGs; M04 was enriched in cells involved in neurogenesis, endothelial cells, and polydendrocytes, whereas M07 was enriched in all interneurons (highest in Vip) and neurogenesis cells. Among the nodes associated exclusively with the source DAG, M10 was enriched in mural and endothelial cells, M01 was enriched in microglia and endothelial cells, M47 was enriched in CA3 pyramidal neurons and endothelial cells, and M05 was enriched in CA1|CA2 pyramidal neurons. M12 was not enriched in any cell-type. To determine if the source- and sink-associated nodes are predictive of the epileptic phenotype, we fitted a decision tree classifier^27^ to the eigengene representing these nodes (Table S6). M07 predicted the withheld control and epilepsy samples with 98% accuracy. Furthermore, the genes of node M07 were significantly overlapped with predictive genes (P = 5.46 × 10^–3^) and gene sets involving epileptic seizures (P = 7.89 × 10^−4^), clonic seizures (P = 2.84 × 10^−3^), and myokymia with neonatal epilepsy (q = 7.91 × 10^−3^).

Taken together, these results showed that the Bayesian network organized the biological events observed across control and epileptic samples into a coherent and directional graph. Although these events occurred in the context of TLE, the direct association of the source and sink nodes with seven DAG nodes suggests that cell communication, GABA neuron differentiation, extracellular matrix, and transcription regulation are more proximal and potentially causal or consequential with respect to TLE. Moreover, the cell-enrichment analyses of nodes M04 and M07, which were found both in the source and sink DAGs, implicate that the interaction between nonneuronal cells and GABAergic interneurons are potential mediators of the identified causal and consequential events in TLE.

### Known epilepsy drugs and related targets are associated with the source- and sink-associated nodes

**Figure 5:**
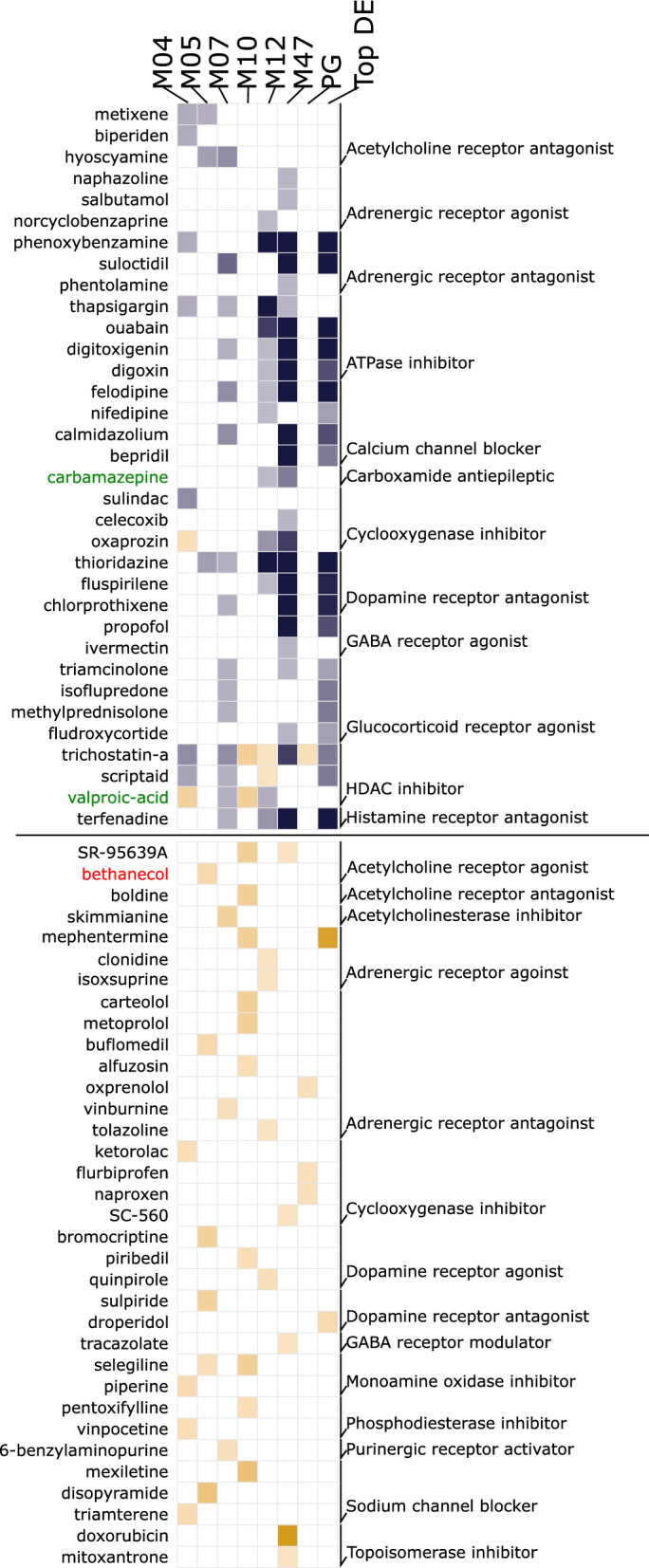
Drugs antagonizing or mimicking the upstream and downstream nodes associated with gene expression profiles. Gene sets of nodes (WGCNA modules) directly connected to source and sink nodes (M04, M05, M07, M10, M12, and M47), predictive gene lists, and top differentially expressed genes (Top DEGs) were used to probe drug-associated signatures from the connectivity map database. Note that known antiepileptic (Carbamazepine and Valproic acid, in green) and epilepsy-inducing (bethanecol, in red) drugs are enriched in nodes directly connected to source and sink nodes, but not in the predictive or top differentially expressed genes. Blue: therapeutic drugs; orange: pro-disease drugs. The − log_10_ of the enrichment q value is plotted. The lighter-to-darker shades of blue and orange represent a lower-to-higher enrichment, respectively. See details in the “Method”s and Tables S7 and S8.

We hypothesized that, if the up- and downstream nodes are directly associated with the epileptic state, drugs that mimic (Fig. 5, bottom panel) or antagonize (Fig. 5, top panel) their gene expression profiles should have epilepsy-inducing or antiepileptic effects, respectively. Therefore, we probed the gene sets associated with the up- and downstream nodes (Table S7) against the connectivity map, a database cataloging signatures of known drugs in diverse cell lines^28^.

Across all seven source- and sink-associated nodes (Fig. 4A,B), 59 mimicking and 212 antagonizing drugs with various modes of action were identified (q < 0.05, Fig. 5, Table S4). Consistent with the known effects of pilocarpine (an acetylcholine receptor agonist) for inducing TLE^29^, SR-95639A and bethanechol^30^, which are drugs with a similar mode of action to that of pilocarpine, were observed as epilepsy-inducing drugs. Similarly, the antiepileptic drugs carbamazepine^31^ and valproic acid^32^ were observed as epilepsy-antagonizing drugs. Carbamazepine was enriched in the M47 node associated with pyramidal neurons (Fig. 4B); valproic acid was enriched in the M07 node associated with interneurons; and carbamazepine and valproic acid were enriched in module M12, which was not associated with any cell type (Fig. 4B). Interestingly, valproic acid had an epilepsy-inducing effect in node M04, associated with oligodendrocytes, and node M10 associated with polydendrocytes, suggesting a diverse mechanism of action across various cell types. Notably, these drugs were not enriched in gene sets associated with predictive genes (Fig. 5, column 7) and DEGs (Fig. 5, column 8), which are commonly used in drug repurposing approaches^33^, indicating that the up- and downstream nodes are linked to the epileptic state.

Regarding drug targets (Table S8), those involved in transcription (topoisomerase), neurotransmission (GABA receptor, norepinephrine reuptake, acetylcholine receptor, dopamine receptor, and monoamine oxidase), ion balance (L-type calcium channel and sodium channel), hormonal balance (estrogen receptor), and signal transduction (mTOR, TP53, and NFkB pathway) were common to epilepsy-inducing and antiepileptic drugs, suggesting that these targets serve as major nodes regulating TLE. The mode of action (and target) unique to epilepsy-inducing effects involved nuclear receptor agonists (retinoid receptor and constitutive androstane receptor), metabolic control inhibitors (thyroid peroxidase, antifolate, dehydrogenase, and carbonic anhydrase), processes involving inhibition of immunomodulation (atherogenesis and cytokine production), and inhibition of signal transduction (PKC and PKA). Similarly, unique targets to epilepsy antagonizers involved homeostasis through hormonal balance (estrogen, steroid, corticosteroid, prolactin, insulin, progestogen, and thyroid), inhibition of cellular processes (protein synthesis, TNF production, DNA replication, DNA synthesis, RNA synthesis, dopamine uptake, and cell proliferation), and inhibition of proliferative and detoxifying enzymes activity. Some known medications (psychoactive, laxative, local anesthetic, antiarrhythmic, selective serotonin reuptake inhibitors, anti-inflammatory, tricyclic antidepressant, and thiazide diuretic drugs) also exhibited a potential epilepsy-antagonizing effect.

Together, the drug-based perturbation studies validate our Bayesian inference, and the associated mode of action suggests that the epilepsy induction involves the nuclear receptor-mediated facilitation of gene expression, as well as the inhibition of immune modulation, and metabolic control, whereas the therapeutic effects mostly involve suppression of these processes.

## Discussion

Unraveling the complex biological disturbances in epilepsy is a major challenge. We investigated the complex phenotype of TLE at multiple levels by combining the available single-cell RNAseq data from the mouse hippocampus with an existing transcriptomic dataset for pilocarpine-induced TLE, originally used to find RNA-editing^34^ sites and develop a computational framework for identifying pharmacological targets^14^. Unlike previous work, our approach is focused on identifying ontologies and druggable mechanisms at cellular resolution. Here, we first showed that there were obvious differences between the functionalities associated with downregulated and upregulated genes (Fig. 1C). The downregulated genes were mostly associated with altered cell–cell signaling, whereas the upregulated genes were associated with increased homeostasis, processes involving the extracellular matrix, and immune and defense responses. Furthermore, we showed that the subtle-to-moderate changes in gene expression associated with astrocytes and pyramidal cells of the dentate gyrus and hippocampal subfields can predict most forms of epilepsy. Second, using deconvolution analyses, we showed that different cell types exhibited disparate changes in cell proportion (Fig. 2C) and that the overall functional alteration had different cellular effects (Fig. 3B). Third, using a Bayesian network of the modules characterized for pathways and cell types (Fig. 4), we identified several potential upstream (causal) and downstream (consequential) mechanisms associated with TLE. Furthermore, as suggested by the enrichment of markers associated with seizure and clonic seizures, these nodes, unlike the predictive gene sets (representing familial forms of epilepsy), represented status epilepticus. Finally, in a separate in silico analysis using gene expression profiles, we showed that drugs with both epilepsy-inducing and -antagonizing effects were associated with causal and consequential nodes (Fig. 5). Collectively, our integrative transcriptomics approach provided novel insights into the cellular pathologies associated with TLE and streamlined a drug discovery/repositioning approach targeting the probable TLE causing nodes.

We observed TLE-specific changes at two levels. At the macroscale, the sparse-classifier-based predictive gene set revealed subtle changes enriched in the astrocyte, dentate gyrus, and hippocampal subfields. Notably, these changes were enriched in various forms of epilepsy (but not seizures)^35,36^ and suggest the involvement of a canonical structural substrate in TLE. Further support regarding the involvement of these structures stemmed from the variable cell-proportion observed in the deconvolution analysis, which relies on expression of discreet gene markers— variability in which is indicative of variable cell states, by means of transcriptional regulation^37,38^ observed in the highest lfc bin (Fig. 1C). Notably, although the deconvolution analysis suggests variable proportions of cell types, an increased proportion of neurons, which are postmitotic cells that seldom divide, is questionable. However, the increased cell proportion may be associated with other cell-level covariates that regulate gene expression, including cell size and activity^39^, which were not considered in our analysis. Furthermore, deconvolution analysis, consistent with the known loss of interneurons in TLE^40^, revealed a reduced proportion of Sst- and Pvalb-positive interneurons. However, Vip-positive interneurons were increased (Fig. 2C), suggesting a role for these interneurons in increasing the epileptic excitotoxicity via its disinhibitory function^41^. Vip-positive interneurons selectively receive acetylcholine and serotonergic afferents^42,43^ and play a role in the long-term dilation of blood micro vessels^44^. Thus, increased activity of these neurons is suggestive of a homeostatic vascular dilation triggered by distant input events.

At the neural circuit level, the Bayesian network, which indicated the triggering of seizures via diverse mechanisms, enumerated many potential upstream causal nodes. However, node M04, which was enriched in components of the blood–brain barrier (endothelial cells, oligodendrocytes, and neurogenesis cells)^45,46^ and node M07, which was enriched in interneurons, were identified as both causal and consequential nodes, possibly suggesting an activity (seizure)-dependent oscillation between the node elements, leading to seizure amplification. Furthermore, a perhaps stronger support for causal associates of nodes M04 and M07 stemmed from the significant enrichment of valproic acid, a potent AED^32^, in the genes associated with these nodes (Fig. 5). Although more comprehensive experimental studies are required to understand the cross talk between interneurons (M07) and nonneuronal cells (M04) in the seizure phenotype, the interneuron-specific preference of oligodendrocytes^47^, activity-dependent oscillatory behavior of oligodendrocytes^48^, seizure-like activity in impaired endothelial cells^49^, antiepileptic role of histone deacetylase inhibitors targeting oligodendrogenesis^50^, and accelerated myelination (function of oligodendrocytes) in the promotion of seizures^51^ are known. Accordingly, our analysis revealed two crucial elements in TLE: a canonical structure involved in TLE, possibly representing the epileptic focus; and a microcircuitry node involving elements of the BBB and interneurons in the potential amplification of seizures.

Causal associations are usually validated using perturbation studies^52^. As these critical data were lacking, a final set of in silico analyses were conducted to utilize gene expression signatures from drug perturbation databases. While validating our Bayesian inference, we observed that known drugs with epilepsy-inducing or epilepsy-antagonizing effects were associated with causal and consequential nodes. Furthermore, on examining the mode of action associated with each drug, we differentiated the key nodes defined by their potential to trigger an epilepsy-inducing or epilepsy-reversing effect. Interestingly, together with the aforementioned biological processes (transcription regulation^53^ and distant serotonergic and cholinergic inputs^54^), we observed other processes involving hormonal balance^55^ and signal transduction through mTOR^56^ and L-type sodium channels^57^ reported in other forms of epilepsy. Finally, the modes of action associated with therapeutic drugs were mostly associated with the inhibition of homeostatic, proliferative, and detoxification processes, suggesting that TLE largely comprises maladaptive processes. Therefore, the increased activity of Vip-positive interneurons suggests a maladaptive dilation of the vascular system, which could initially be triggered to maintain blood flow to meet the increased TLE-associated metabolic demands.

In conclusion, TLE is a system level phenomenon with maladaptive, amplified, and nonlinear interactions between different system components. Amidst this complexity, finding a precise therapeutic drug requires knowledge of predictive, causal, and consequential changes at cellular resolution. To that effect, the integrative data-analysis approach used here not only reveals key nodes associated with TLE (and other forms of epilepsy) and seizure but also demonstrates a novel workflow to identify targeted therapeutics.

## Methods

### Data

This study used data previously collected by^14^, who performed high-throughput RNA sequencing on whole hippocampus samples from pilocarpine-treated (n = 108) and littermate control (n = 103) adult (> 12 weeks) male Crl:NMRI(Han)-FR mice. Pilocarpine-treated (injected intraperitoneally; 300 mg/kg) mice developed convulsive seizures and effectively displayed status epilepticus (SE)^14^. Modeling epilepsy, the surviving mice showed spontaneous recurrent seizures. 28 days following SE the hippocampus was extracted for RNAseq analysis. Fastq.gz files for all samples were downloaded from the European Nucleotide Archive (accession number: PRJEB18790) and were aligned to the mouse reference genome GRCm38 (provided by Ensembl) using HISAT2 aligner. Count data were generated for the reads aligned to exons and transcripts using GenomicFeature and GenomicAlignments^58^ in R and the mouse gene model (GTF file) provided by Ensembl.

### Differential expression analysis

After filtering out genes present in a minimum of 100 samples, 15,300 genes (Table S1) were examined for differential expression between the control and epileptic groups using Deseq2 in R. To segregate the effects of subtle and obvious changes, all differentially expressed genes (DEGs) (false discovery rate [FDR]-corrected P value < 0.05) were binned based on their log-fold change (Fig. 1B). To filter out the minimal set of genes that could discriminate the epileptic phenotype from the control phenotype, we used a sparse classification strategy employing nearest shrunken centroids^59^ and the diagonal discriminant classifiers^60^ implemented by MLseq in R^61^. The classification model identified 119 genes (termed predictive genes, Table S3) that were segregated as being up- or downregulated based on their initial Deseq2-based log-fold change (Table S1). To characterize the predictive genes, we searched for their enrichment in gene ontology (GO) terms, different log-fold change bins of DEGs, and cell type-specific discriminant markers. In all enrichment analyses, we used the hypergeometric overlap analysis implemented by GeneOverlap in R. A background of 21,196 genes and a significant cut-off q value < 0.05 were used.

### Pathway enrichment analysis

To identify the biological pathways affected at the different log-fold change bins of DEGs, we searched for the enrichment of different GO terms in the Biological Pathway (GOBP), Molecular Function (GOMF), and Cellular Component (GOCC) categories using the GO database. Only fdr corrected pathways (q value < 0.05) were considered for subsequent analysis. To reduce and catalog our long list of GO terms into an interpretable format, we clustered the pathways based on biological themes^62,63^. Briefly, the theme for a given pathway was selected based on either a text search, in which the name of the theme was used as a keyword for text query, or on the parent–child association between the GO terms in our list of significant pathways (child pathways) and the handpicked pathways in the GO database (parent pathway) representing the theme using GOdb in R.

### Cellular deconvolution analysis

To estimate the TLE-associated changes in cell type proportion and the genes and pathways affected by an altered cell type proportion, we employed the previously described deconvolution analysis^17,19,63,64^ in the bscqsc package in R. The analysis involved four steps.

#### Identifying cell type-specific marker genes

Using the single-cell RNAseq dataset for mouse hippocampus available from the Gene Expression Omnibus (accession: GSE116470)^18^, we identified clusters of cell types (Fig. 2A) and markers that were specific to each cluster (Figure S2, Table S4) using SEURAT in R for single-cell analysis^65^. To segregate cell clusters based on subtle differences in expression, the resolution parameter was set to 1.2.

#### Building the reference signature matrix of marker genes (Figure S2)

This matrix contained the highly discriminatory marker gene expression of each cell-type cluster averaged across all cells of a given cluster. A gene was considered cluster-specific only when it showed a twofold difference in expression compared with that in other cell-type clusters.

#### Estimating proportions

The resulting reference signature matrix was used to estimate the cluster-specific cell type proportion in control and epileptic samples using Support Vector Regression (SVR) implemented by CIBERSORT in R^19^. The Wilcoxon test was used to estimate the significance of altered cell-type proportions using stat_compare_means function in R. The Bonferroni correction was performed using the p.adjust.method parameter within the stat_compare_means function set to "BH".

#### Validating the deconvolution approach

To validate the estimated cell-type proportion, we deconvolved the control and epileptic data using various other existing deconvolution techniques implementing non-negative least square (NNLS)^20^, reweighted least square (RLS)^21^, and linear mixed model (LMM)^22^ based algorithms. For this, we used the granulator package in R, which provides a uniform testing interface to benchmark many deconvolution methods. The estimated proportion from various algorithms were correlated with the proportion estimated using SVR based method described above. The mean correlation between the cell-type proportion estimated (diagonal of correlation matrix Figure S3, top) by different algorithm was used to measure their agreement with SVR based method on assigning cell-type specific signals. Notably, the reweighted least squares (RLS) method deconvolved the cell proportion in the highest agreement with the SVR based approach. However, as RLS showed several negative proportions (Figure S3, bottom), for all subsequent analysis, SVR based proportions were used.

#### Adjusting the bulk tissue gene expression for differences in cell-type proportion

To assess the effect of altered proportions of different cell types, we statistically regressed out the effect of cell-type clusters representing pyramidal neurons, interneurons, and non-neuronal cells that showed significant changes in proportion in TLE vs. control samples. This was implemented by expanding the design model used for examining the differential expression between TLE and control subjects by incorporating the estimated cell-type proportion (from step C) in the design matrix. Genes with further reduced P-values (Table S5) were considered as being affected by the altered cell-type proportion and were applied to a pathway analysis (Fig. 3B) using the GO database.

### Bayesian network analysis

To identify the probabilistic upstream causal and downstream consequential associations between TLE and gene coexpression modules representing different biological themes, we used a weighted gene coexpression network analysis (WGCNA)^66^ and Pigengene^67^ in R. For each cohort, count data that were normalized based on the size factor (using DESeq2 in R) were used to construct a matrix of pairwise Pearson’s correlations between genes, which was then transformed to a signed adjacency matrix using a power of β = 12. Adjacency matrices provide the connectedness between genes. To calculate the interconnectedness between genes (which defines “modules”), we derived the topological overlap, a biologically meaningful measure of the similarity between two genes based on their coexpression relationship with all other genes^68^. Consensus modules between the control and epilepsy cohorts were then identified using the blockwiseConsensusModules function. Fifty consensus modules numerically labeled 1–50, were functionally characterized using the GO database for the GOBP, GOMF, and GOCC GO categories; the top representative pathways with a Bonferroni-corrected P value of < 0.05 (Table S6 were used to label the modules. Modules that were not characterized and module 0, which comprised genes not assigned to any module^69^, were not analyzed further.

The first principal component (the eigengene) summarized a given module by accounting for the maximum variability of all its constituent genes^25^ and was used as a biological signature^27^ of the module and to identify the mechanisms associated with TLE. We used Pigengene in R, which works on this principle using the module eigengenes as random variables to train a Bayesian network by modeling the probabilistic dependencies between all modules as directed acyclic graphs (DAGs, Fig. 4A,B; modules in the DAG network are termed “nodes”). In addition, the network had a categorical variable that modeled the disease state. Its value corresponded to the labels associated with the samples, i.e., control and epilepsy. To simplify the inference, the disease node served either as a source (with no parent terms above it) or as a sink (with no child terms below it)^63,67^. This was implemented by blacklisting all incoming and outgoing edges to the source and node, respectively. To search for the optimal Bayesian network fitting our data, we performed 1000 permutations using the default parameters in Pigengen.

### Drug target enrichment analysis

To identify causal/consequential nodes associated with epilepsy-inducing and therapeutic drugs, we first segregated the up- and downregulated genes (Table S7) associated with the nodes (WGCNA modules) that were directly connected to source and sink nodes (Fig. 4A,B, nodes in blue) based on their log-fold change estimates derived from the differential expression analysis (Table S1). Next, drug-specific gene markers (associated with the connectivity map database^28^) that were attributed to up- and downregulated effects were downloaded from the Enricher library of gene sets^16^, and significant enrichment of drug-induced molecular signatures in the gene sets associated with causal and consequential nodes (Table S7) were calculated using hypergeometric overlap analysis. Based on the signature-reversing principle^70^, an enrichment in the inverse direction (i.e., up- or downregulated drug signature enriched in down- or upregulated WGCNA module genes, respectively) was considered therapeutic, whereas an enrichment in the similar direction (i.e., up- or downregulated drug signature enriched in up- or downregulated WGCNA module genes, respectively) was considered disease inducing. Gene markers of drugs with known modes of action and targets were used to understand druggable mechanisms (Table S8) and targets.

## Supplementary Information

Supplementary Information 1.

Supplementary Information 2.

Supplementary Information 3.

Supplementary Information 4.

Supplementary Information 5.

Supplementary Information 6.

Supplementary Information 7.

Supplementary Information 8.

Supplementary Information 9.

Supplementary Information 10.

Supplementary Information 11.

Supplementary Information 12.

Supplementary Information 13.

## Data Availability

The datasets used in this study are available in the following databases: RNA-Seq data: European Nucleotide Archive (ENA; http://www.ebi.ac.uk/ena) under accession number PRJEB18790. Single-cell RNA-seq data: Gene Expression Omnibus https://www.ncbi.nlm.nih.gov/geo/query/acc.cgi?acc=GSE116470.
