# Supplementary material for: Cellular, molecular, and therapeutic characterization of pilocarpine-induced temporal lobe epilepsy
Source: Sci Rep. 2021 Sep 27;11:19102. doi: 10.1038/s41598-021-98534-3 (PMC8476594; doi:10.1038/s41598-021-98534-3)
Supplement: Supplementary file 2 — Supplementary Information 2. [file 41598_2021_98534_MOESM2_ESM.pdf]

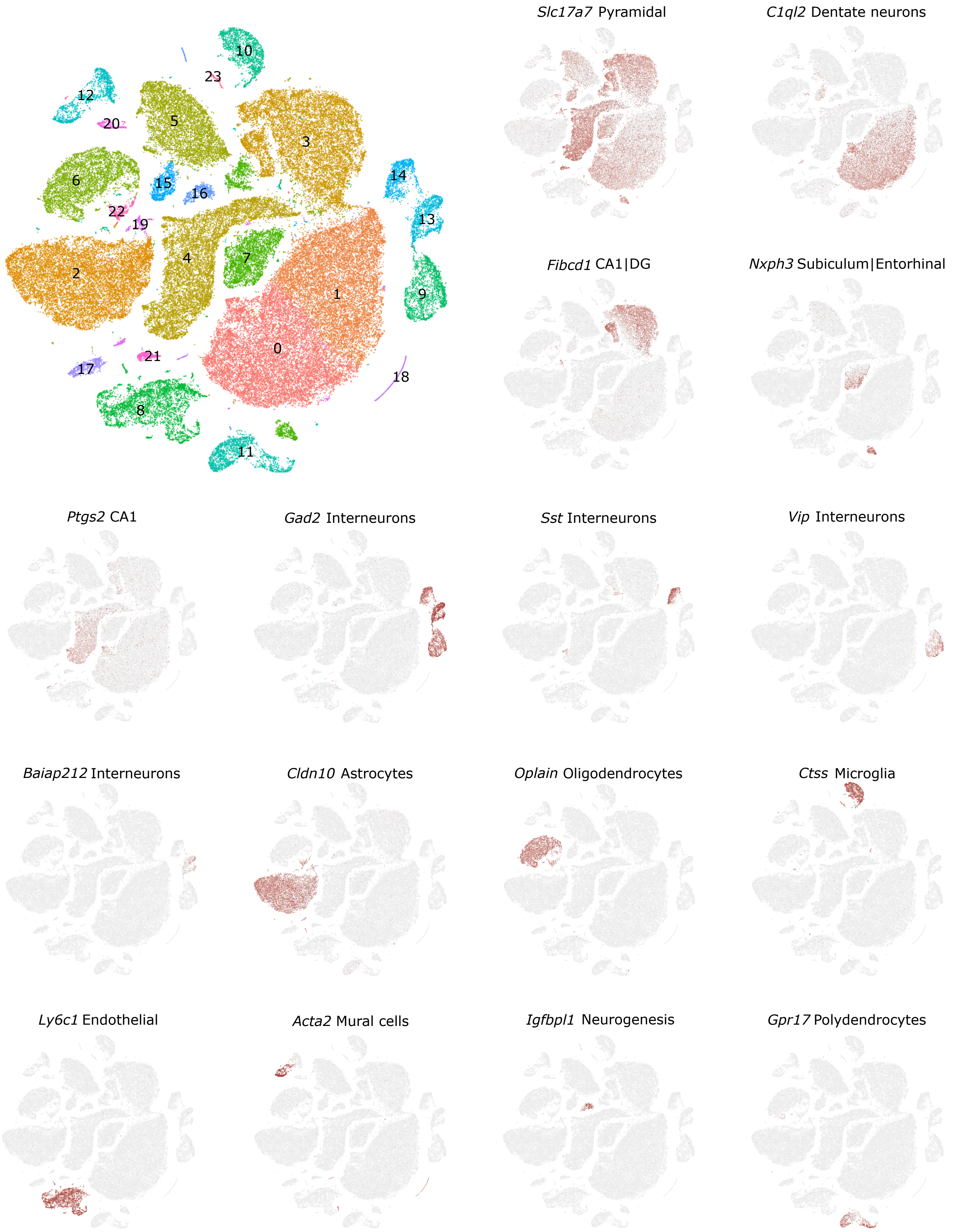

10
23
12
20
5
3
6
15
16
14
22
13
19
4
7
2
1
9
0
21
18
17
8
11
Slc17a7 Pyramidal
C1ql2 Dentate neurons
Fibcd1 CA1|DG
Nxph3 Subiculum|Entorhinal
Ptgs2 CA1
Gad2 Interneurons
Sst Interneurons
Vip Interneurons
Baiap212 Interneurons
Cldn10 Astrocytes
Oplain Oligodendrocytes
Ctss Microglia
Ly6c1 Endothelial
Acta2 Mural cells
Igfbpl1 Neurogenesis
Gpr17 Polydendrocytes

Figure S1a
