# Supplementary material for: Cellular, molecular, and therapeutic characterization of pilocarpine-induced temporal lobe epilepsy
Source: Sci Rep. 2021 Sep 27;11:19102. doi: 10.1038/s41598-021-98534-3 (PMC8476594; doi:10.1038/s41598-021-98534-3)
Supplement: Supplementary file 3 — Supplementary Information 3. [file 41598_2021_98534_MOESM3_ESM.pdf]

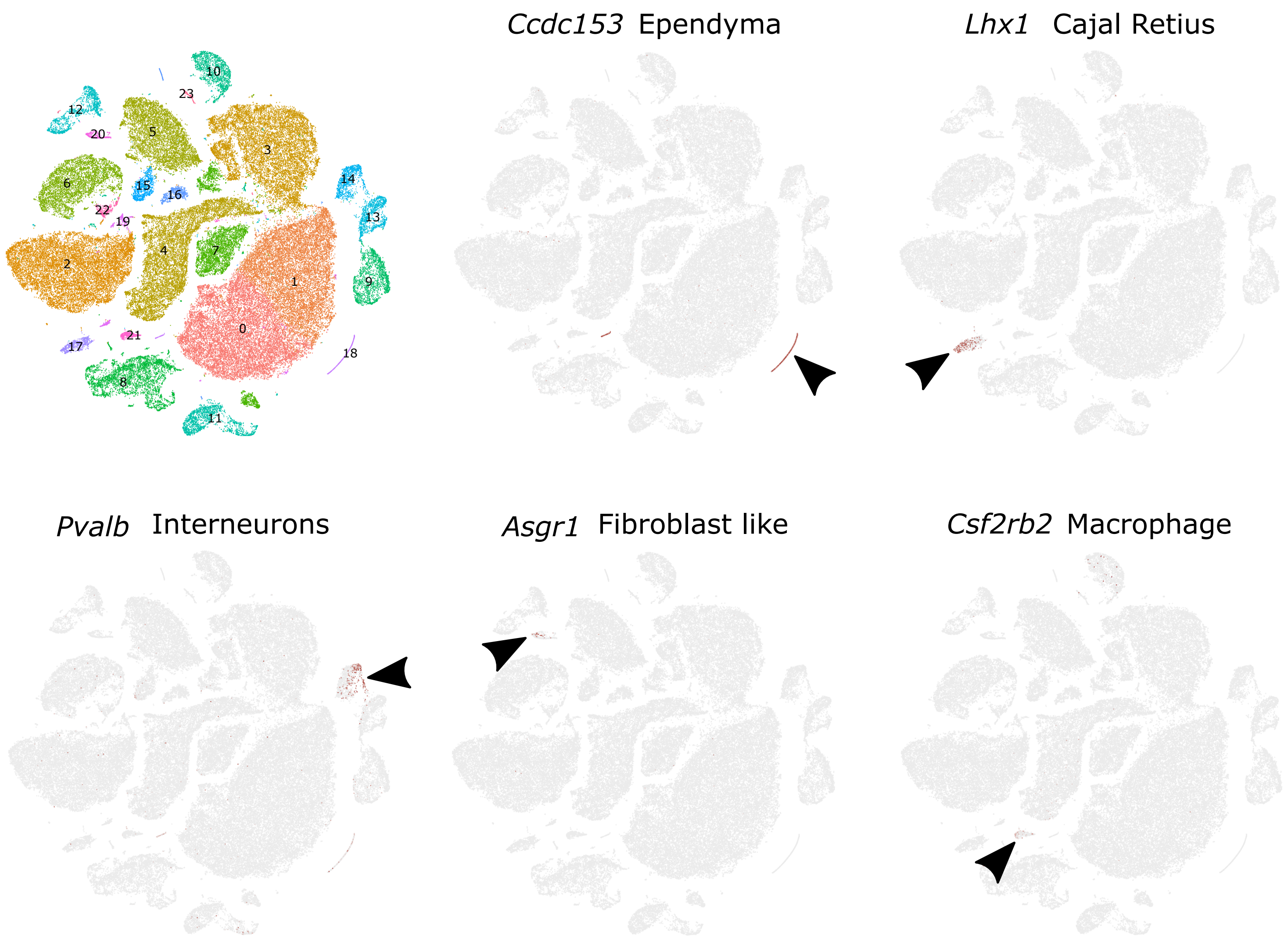
Ccdc153 Ependyma
Lhx1 Cajal Retius
Pvalb Interneurons
Asgr1 Fibroblast like
Csf2rb2 Macrophage

Figure S1b
