# Supplementary figures and images for: Cellular, molecular, and therapeutic characterization of pilocarpine-induced temporal lobe epilepsy
Source: Sci Rep. 2021 Sep 27;11:19102. doi: 10.1038/s41598-021-98534-3 (PMC8476594; doi:10.1038/s41598-021-98534-3)

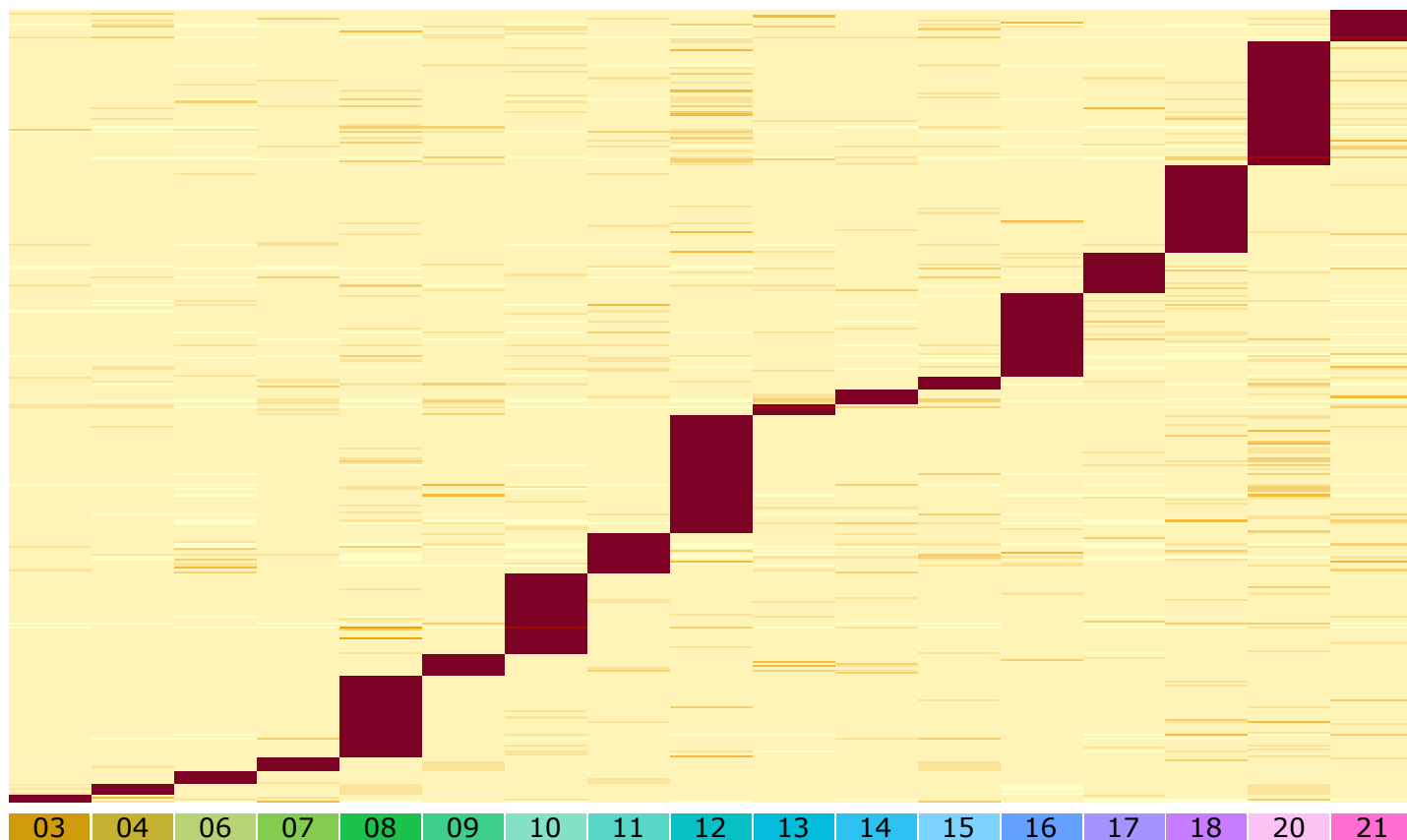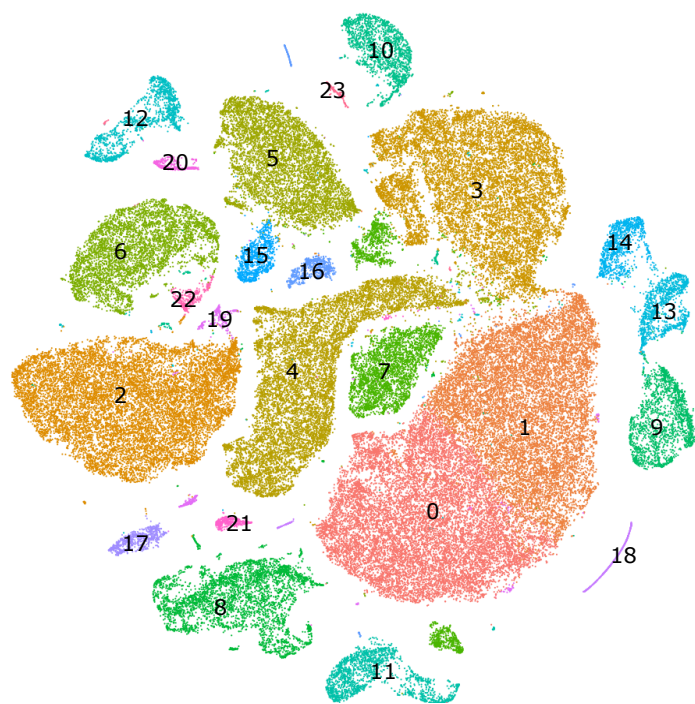

Figure S2

Supplement: Supplementary file 4 — Supplementary Information 4. [file 41598_2021_98534_MOESM4_ESM.pdf]

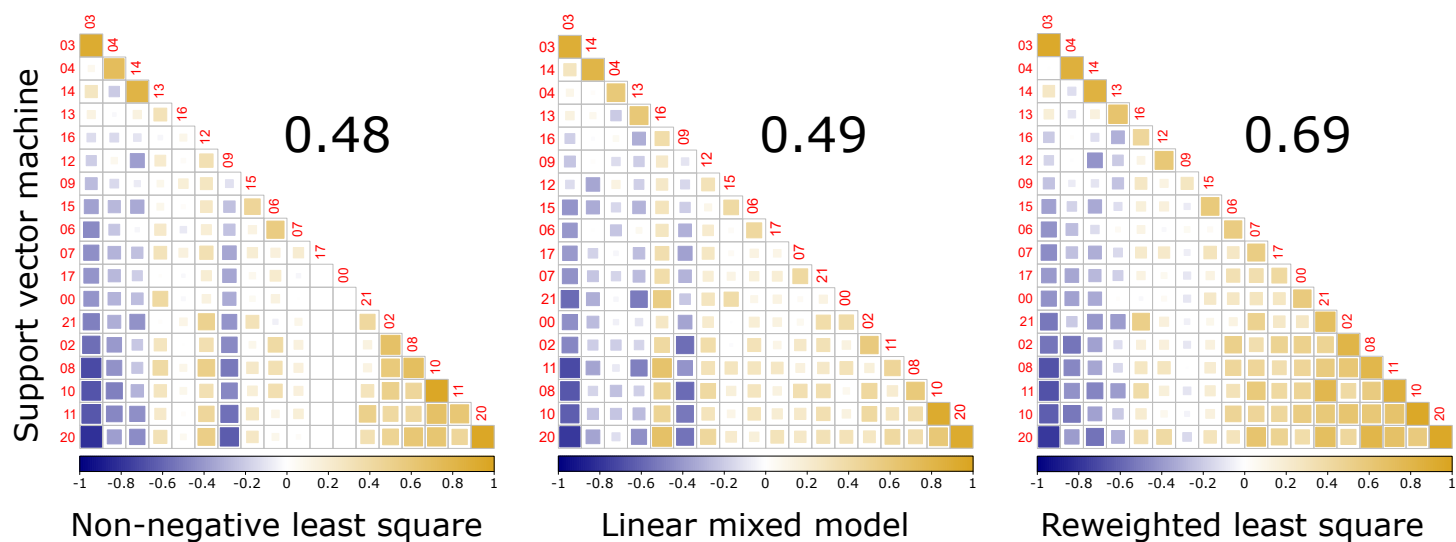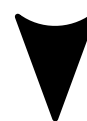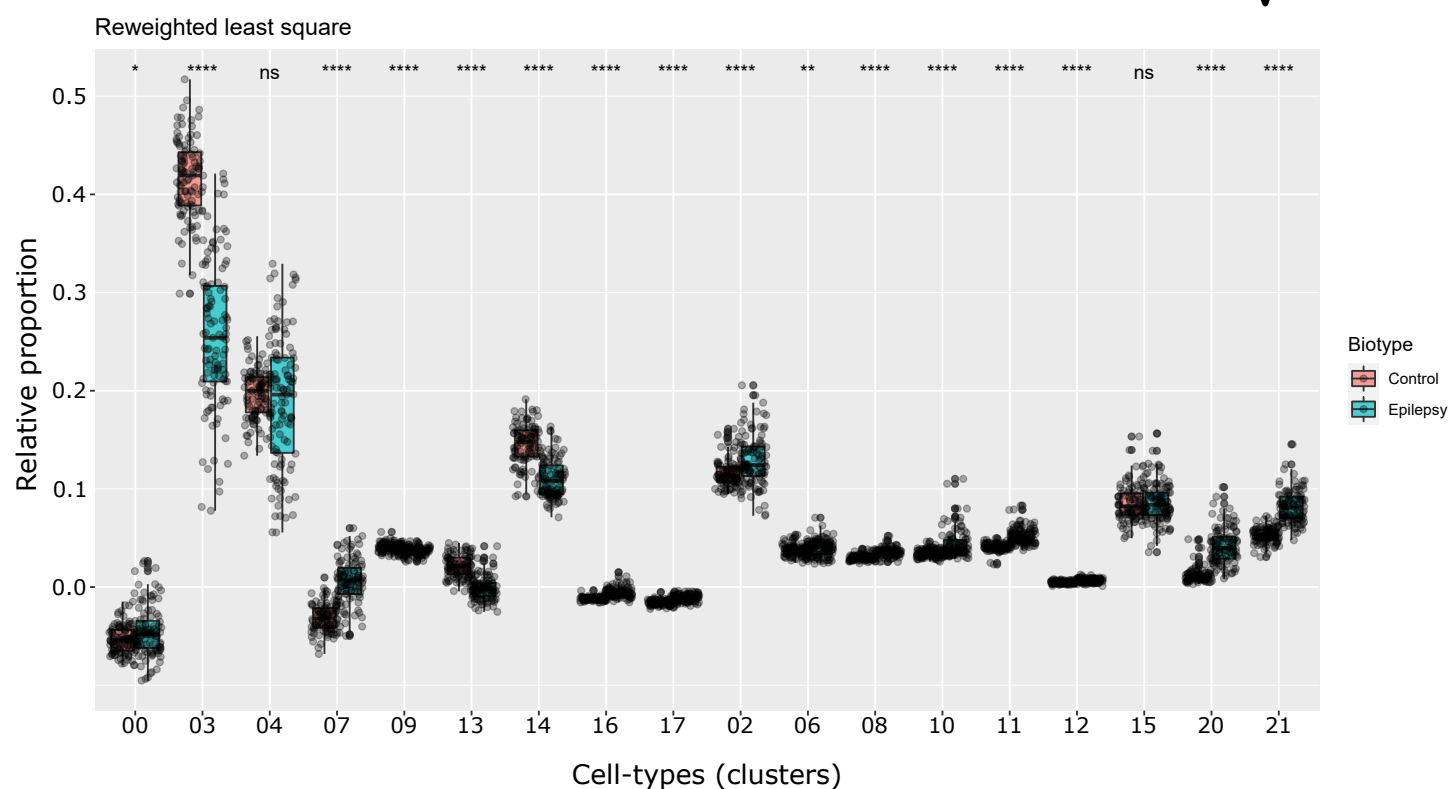

Figure S3

Supplement: Supplementary file 5 — Supplementary Information 5. [file 41598_2021_98534_MOESM5_ESM.pdf]
